# Wasabi Component 6-(Methylsulfinyl)hexly Isothiocyanate and Derivatives Improve the Survival of Skin Allografts

**DOI:** 10.3390/ijms23158488

**Published:** 2022-07-30

**Authors:** Tun-Sung Huang, Chih-Jung Ko, Jiunn-Chang Lin, Ming-Ling Hsu, Chun-Chuan Ko, Chih-Wen Chi, Tung-Hu Tsai, Yu-Jen Chen

**Affiliations:** 1 Institute of Traditional Medicine, National Yang Ming Chiao Tung University, Taipei 112304, Taiwan; tshuang@mmh.org.tw; 2 Department of Surgery, MacKay Memorial Hospital, Taipei 10449, Taiwan; jiunn@mmh.org.tw; 3 Department Medical Research, MacKay Memorial Hospital, New Taipei City 251020, Taiwan; bbebella.2220@mmh.org.tw (C.-J.K.); mlhsu@mmh.org.tw (M.-L.H.); waterdragon.b993@mmh.org.tw (C.-C.K.); b514.b514@mmh.org.tw (C.-W.C.); 4 MacKay Junior College of Medicine, Nursing, and Management, New Taipei City 11260, Taiwan; 5 Department of Surgery, MacKay Medical College, New Taipei City 25245, Taiwan; 6 Department of Radiation Oncology, MacKay Memorial Hospital, Taipei 104217, Taiwan; 7 Department of Artificial Intelligence and Medical Application, MacKay Junior College of Medicine, Nursing and Management, Taipei 112021, Taiwan; 8 Department of Medical Research, China Medical University Hospital, Taichung 404332, Taiwan

**Keywords:** wasabi, 6-(methylsulfinyl) hexyl isothiocyanate, transplantation, dendritic cells

## Abstract

We tested the effect of 6-(Methylsulfinyl)hexyl Isothiocyanate (6-MITC) and derivatives (I7447 and I7557) on the differentiation and maturation of human myeloid dendritic cells (DCs) in vitro, and skin transplantation in vivo. Triggering of CD14^+^ myeloid monocyte development toward myeloid DCs with and without 6-MITC and derivatives to examine the morphology, viability, surface marker expression, and cytokine production. Stimulatory activity on allogeneic naive T cells was measured by proliferation and interferon-γ production. The skin allograft survival area model was used to translate the 6-MITC and derivatives’ antirejection effect. All of the compounds had no significant effects on DC viability and reduced the formation of dendrites at concentrations higher than 10 μM. At this concentration, 6-MITC and I7557, but not I7447, inhibited the expression of CD1a and CD83. Both 6-MITC and I7557 exhibited T-cells and interferon-γ augmentation at lower concentrations and suppression at higher concentration. The 6-MITC and I7557 prolonged skin graft survival. Both the 6-MITC and I7557 treatment resulted in the accumulation of regulatory T cells in recipient rat spleens. No toxicity was evident in 6-MITC and I7557 treatment. The 6-MITC and I7557 induced human DC differentiation toward a tolerogenic phenotype and prolonged rat skin allograft survival. These compounds may be effective as immunosuppressants against transplant rejection.

## 1. Introduction

Transplantation remains the most efficient solution to treat end-organ disease; however, graft rejection after transplant is a major cause of graft loss and death. After the introduction of cyclosporine to patients receiving organ transplants in 1978, calcineurin inhibitors (CNIs) became the mainstay of immunosuppressants to prevent graft rejection. Cyclosporin and tacrolimus are potent immunosuppressants and have considerably expanded the field of allogeneic organ transplantation. Tacrolimus and cyclosporin, which target the FK-binding protein and cyclophilin, respectively, inhibit the phosphatase activity of calcineurin and the nuclear factor of T-cell activation [1]. The inhibition of T-cell proliferation also indicates that immunity against an implanted graft is inhibited. CNI treatment results in side effects, including nephrotoxicity and neurotoxicity, which must be managed carefully to avoid toxicity [2]. Some strategies have included the conversion of CNIs to mammalian target of rapamycin (mTOR) inhibitors to improve renal function, but this may increase the risk of acute rejection [3]. Although immunosuppressive agents have succeeded in reducing the incidence of graft rejection and early graft loss, they are associated with serious complications, such as a high risk for opportunistic infection and de novo malignancy [4].

The process of graft rejection includes alloantigen recognition, cytotoxic T-cell activation, clonal expansion, and organ inflammation. Dendritic cells are potent antigen-presenting cells that bridge the innate and adaptive immunity [5]. They play important roles in both immunity and tolerance. The mature dendritic cells (DCs) present antigens to effector T cells to induce an immunostimulatory response, whereas immature DCs expand the regulatory T cells to induce immunotolerance [6,7,8,9]. The functional dichotomy of dendritic cells depends on the maturation status and the surrounding microenvironment. An ideal clinical immunosuppressant would promote immature dendritic cell status and subsequently induce T regulatory cell expansion, resulting in the immune tolerance of the transplanted organ [5].

The natural product, 6-(methylsulfinyl)hexyl isothiocyanate (6-MITC), was isolated from Wasabia japonica (wasabi), a popular spice used in Japanese food worldwide. This compound has anti-inflammatory [10], anti-microbial, and anti-cancer [11,12] properties. Furthermore, 6-MITC may prevent the complications of opportunistic infection and de novo malignancy following transplant. The synthetic compounds derived from 6-MITC include 6-(methylsulfenyl)hexyl isothiocyanate (I7447) and 6-(methylsulfonyl)hexyl isothiocyanate (I7557). They were synthesized by the chemical modification of the 6-MITC sulfone moiety by the deletion and addition of an oxygen atom, respectively [13]. The 6-MITC induces G2/M phase arrest in cancer cells and is capable of blocking the KRAS/BRAF and PI3K/AKT signaling pathways [11,12]. Because the effect of these compounds on alloantigen recognition and T-cell activation are unknown, we established an experimental skin allograft model as a drug screen for immunosuppressants following transplantation [14,15,16].

In this study, we determined the effect of 6-MITC and its derivatives on DC differentiation and maturation in vitro. A rat-skin transplantation model was established to examine the immunomodulatory effect of 6-MITC and its derivatives in vivo.

## 2. Results

### 2.1. Morphological Features of 6-MITC and Its Derivatives on DCs

**Figure 1:**
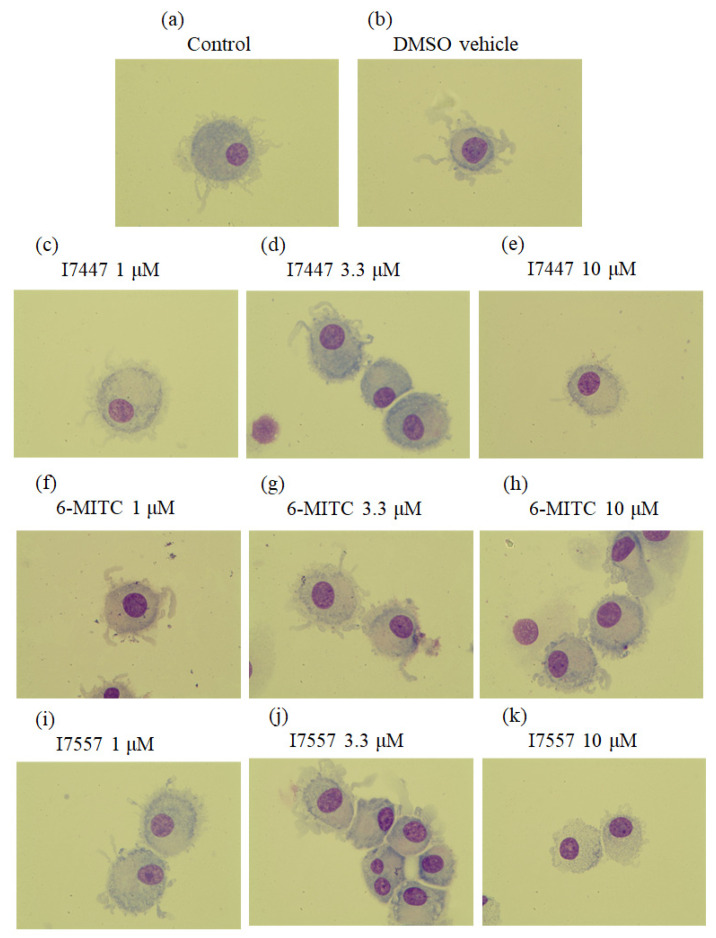
Morphological observation of dendritic cells. CD14+ monocytes were treated with (**a**) control; (**b**) vehicle control DMSO; (**c**–**e**) I7447; (**f**–**h**) 6-MITC; or (**i**–**k**) I7557 for 7 days to generate dendritic cells. Cells were stained by Liu’s method for morphological examination by light microscopy (magnification 1000×). As the concentration of 6-MITC, I7457, and I7557 increased, the dendrites of the cells were reduced and shorter.

The monocyte-derived DCs exhibited typical morphological characteristics of mature DCs after stimulation with LPS, including nonadherence, the presence of many sharp cytoplasmic projections, and an abundant cytoplasm. As the concentration of the 6-MITC, I7457, and I7557 increased, the dendritic projections decreased and became shorter (Figure 1), and the cell contour was indistinct. These morphological changes in 6-MITC, I7447, and I7557-treated monocyte-derived DCs suggest an atypical or immature status during DC development. The histogram from the flow cytometric analysis revealed that the average size of the dendritic cells progressively decreased as the concentration of 6-MITC, I7457, and I7557 increased.

### 2.2. Effect of 6-MITC and Chemical Derivatives on DC Viability

**Figure 2:**
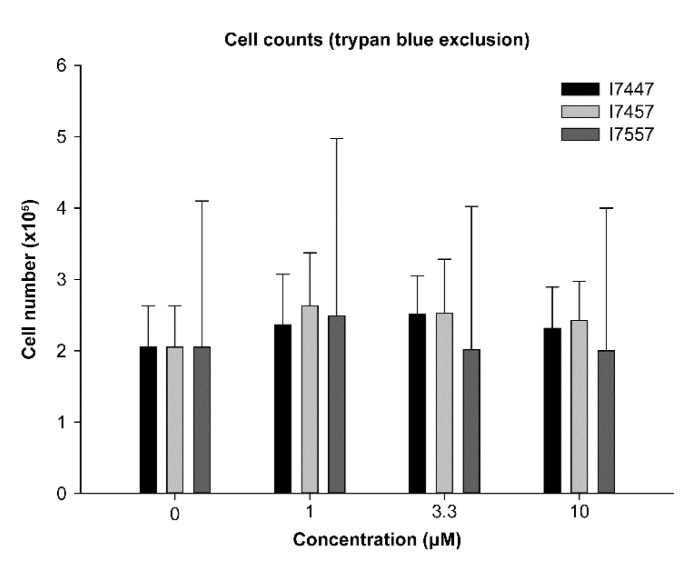
6-MITC, I7447, and I7557 did not significantly affect the viability of dendritic cells at 1, 3.3, and 10 μM.

The 6-MITC, I7447, and I7557 negligibly affected the viability of the cells, as assessed by a trypan blue exclusion test. The viability of the 6-MITC-, I7447-, and I7557-treated dendritic cells were not statistically significantly different (Figure 2).

### 2.3. Modulation by 6-MITC and Its Derivatives on DC Surface Marker Expression

**Figure 3:**
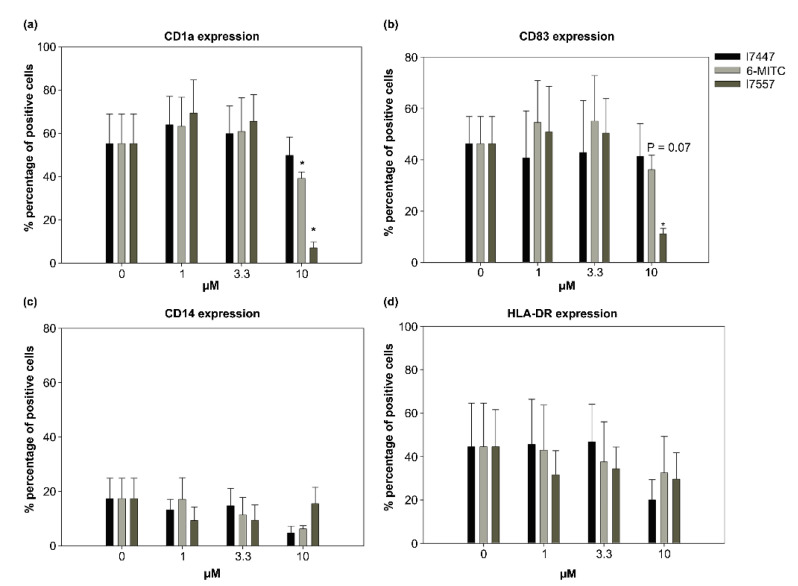
Effect of 6-MITC and its derivatives on the expression of the dendritic cell surface markers, CD1a and CD83. DCs were treated with 6-MITC/I7447/I7557 at concentrations of 0, 1, 3.3, and 10 μM for 7 days. CD1a (**a**), CD83 (**b**), CD14 (**c**), and HLA-DR (**d**) expression was assessed by flow cytometry. (**a**,**b**) The 6-MITC and I7557 exhibited a dual effect on the CD1a and CD83 expression. * *p* < 0.05 in comparison to control.

The 6-MITC and I7557 exhibited a dual effect on the CD1a and CD83 expression. CD1a expression was 55.23 ± 13.68% in the control group, 6-MITC- and I7557-treated CD1a expression was 60.84 ± 15.58% and 65.53 ± 12.37% at 3.3 μM (*p* < 0.05), and 39.12 ± 2.96% and 7.05 ± 2.68% at 10 μM (*p* < 0.05), respectively. CD1a is known as a differentiation marker, whereas CD83 is a maturation marker [17]. The CD83 expression was 38.85 ± 12.14% in the control group, 6-MITC- and I7557-treated CD83 expression was 60.82 ± 14.05% and 53.31 ± 13.64% at 3.3 μM (*p* < 0.05), and 36.58 ± 6.42% (*p* = 0.07) and 11.02 ± 2.34% (*p* < 0.05) at 10 μM, respectively (Figure 3). As shown in Figure 3, the 6-MITC and I7557 dose-dependently increased the CD1a and CD83 expression in the dendritic cells at concentrations of 1 and 3.3 μM. In contrast, the expression was inhibited by the treatment with 10 μM 6-MITC and I7557.

### 2.4. Secretion of Cytokines from DCs in the Presence of 6-MITC and Its Derivatives

**Figure 4:**
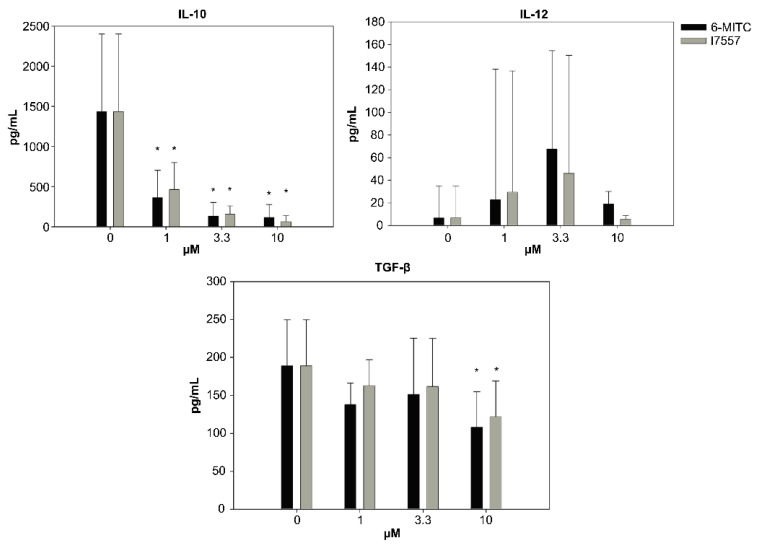
The secretion of IL-10 and TGF-β was inhibited by 6-MITC and I7557 in a dose-dependent manner. * *p* < 0.05 in comparison to control.

The secretion of IL-10 and TGF-β from the dendritic cells was inhibited by 6-MITC and I7557 in a dose-dependent manner. The 6-MITC and I7557 had dual effects on IL-12 secretion, which exhibited a crescendo–decrescendo pattern (Figure 4).

### 2.5. Effect of 6-MITC and I7557 on DC Stimulation of Allogeneic Naive T Cells

**Figure 5:**
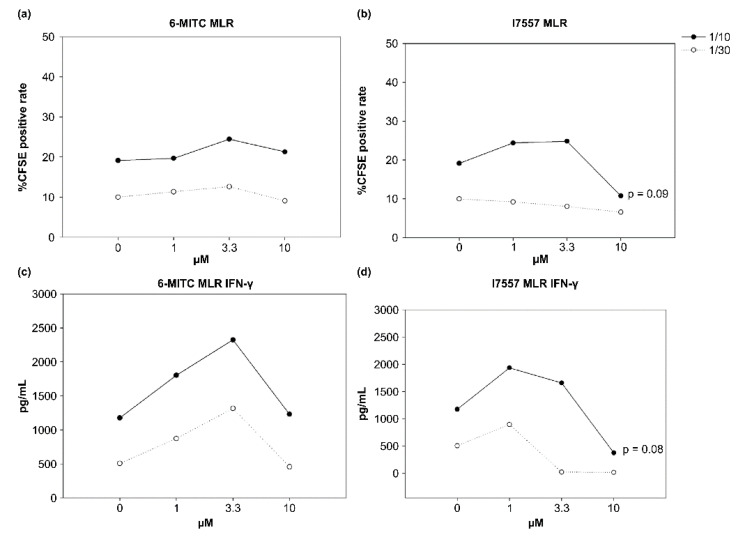
Proliferation and interferon (IFN)-γ secretion of allogeneic CD4^+^CD45RA^+^ naive T-cells stimulated by DCs generated in various cultures. CD4^+^CD45RA^+^ T-cell proliferation stimulated by 6-MITC and I7557-treated DCs as measured by carboxyfluorescein succinimidyl ester (CFSE) uptake. (**a**,**c**) No significant effects between 6-MITC-treated DCs and naïve T-cell; (**b**,**d**) Dual effect between I7557-treated DCs and T-cells.

The 6-MITC showed no significant effects on allogeneic CD4^+^CD45RA^+^ T cells, whereas I7557 had dual effects on the T cells. The low-concentration-treated DCs stimulated the proliferation of allogeneic CD4^+^CD45RA^+^ T cells, whereas the DCs treated with high-doses tended to inhibit the naïve T-cell proliferation (Figure 5). Interferon (IFN)-γ production by these allogeneic naive T cells exhibited a similar curve to that of the T-cell proliferation for the I7557-treated group.

### 2.6. Effect of 6-MITC and I7557 on Allograft Survival

**Figure 6:**
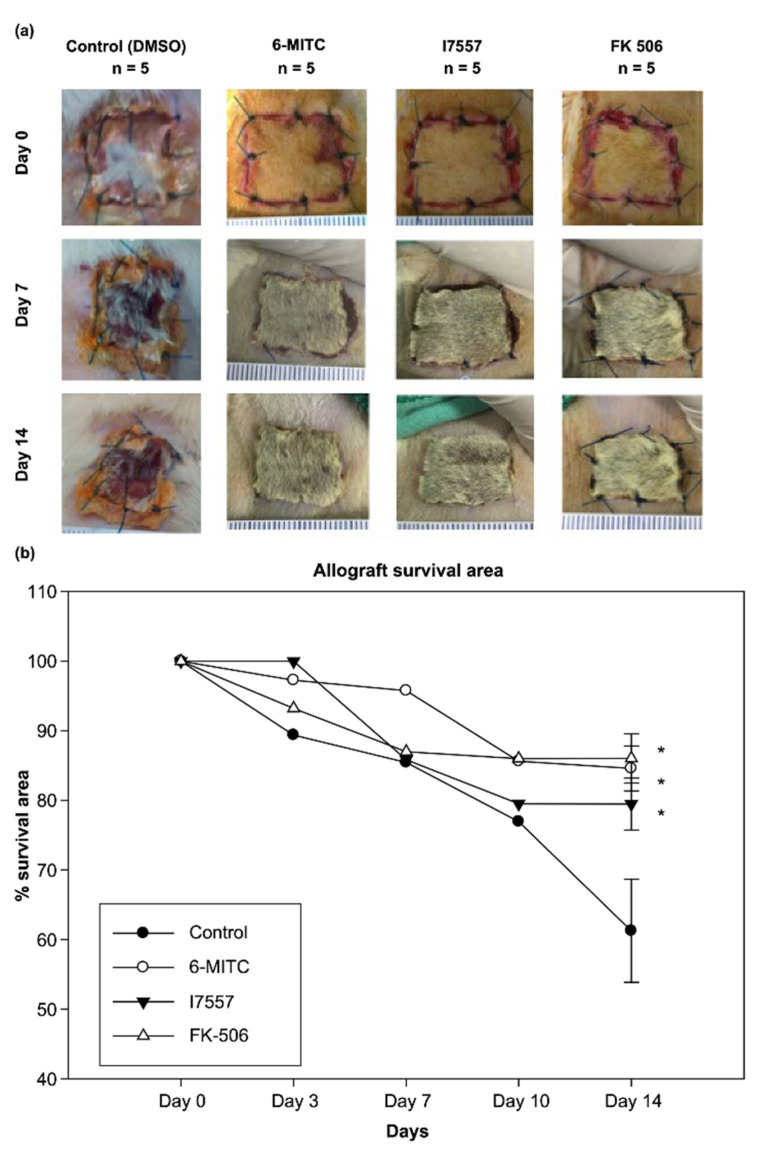
(**a**) 6-MITC and I7557 maintains skin allograft integrity; (**b**) 6-MITC and I7557 prolongs skin allograft survival by maintaining the survival area (* *p* < 0.05).

To assess graft survival, we recorded the area of viable graft in each recipient for 14 consecutive days. A series of images showing the effects of 6-MITC, I7557, and FK506 on the integrity of the skin allografts is shown in Figure 6A. The treatment with 6-MITC and I7557 significantly prolonged the skin graft survival by maintaining the survival area (Figure 6B). The day 7 skin graft survival area (%) was 75.6 ± 6.8% in the control group and 95.7 ± 2.5%, 85.8 ± 4.2%, and 87.0 ± 4.1% in the 6-MITC, I7557, and FK506 groups, respectively. The day 14 skin graft survival area (%) was 61.2 ± 7.4% in the control group and 84.6 ± 3.2%, 79.4 ± 3.7%, and 86.4 ± 3.1% in the 6-MITC, I7557, and FK506 groups, respectively (*p* < 0.05).

### 2.7. Histological Analysis

**Figure 7:**
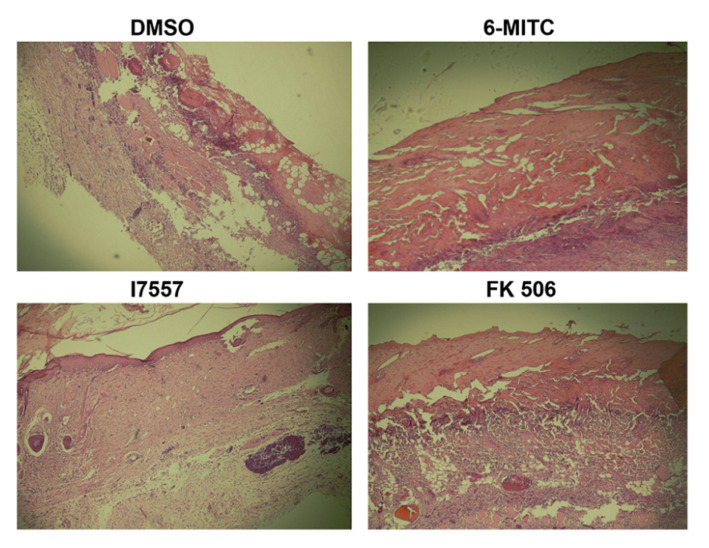
6-MITC and I7557 reduce the inflammatory reaction with improved integrity of the epidermis and less marked inflammatory cell infiltration compared with the DMSO vehicle controls. Magnification 40×.

By day 14, the skin allografts in the dimethyl sulfoxide (DMSO) control group exhibited severe epidermal necrosis and necrotizing vasculitis. The treatment with 5.0 mg/kg 6-MITC or I7557 markedly reduced the inflammatory reaction and showed improved integrity of the epidermis and reduced inflammatory cell infiltration, compared with the DMSO vehicle controls (Figure 7). The positive control, FK-506 (3.0 mg/kg), also showed a superior effect at maintaining epidermal integrity and reducing graft inflammation.

### 2.8. Immunohistochemical Study

**Figure 8:**
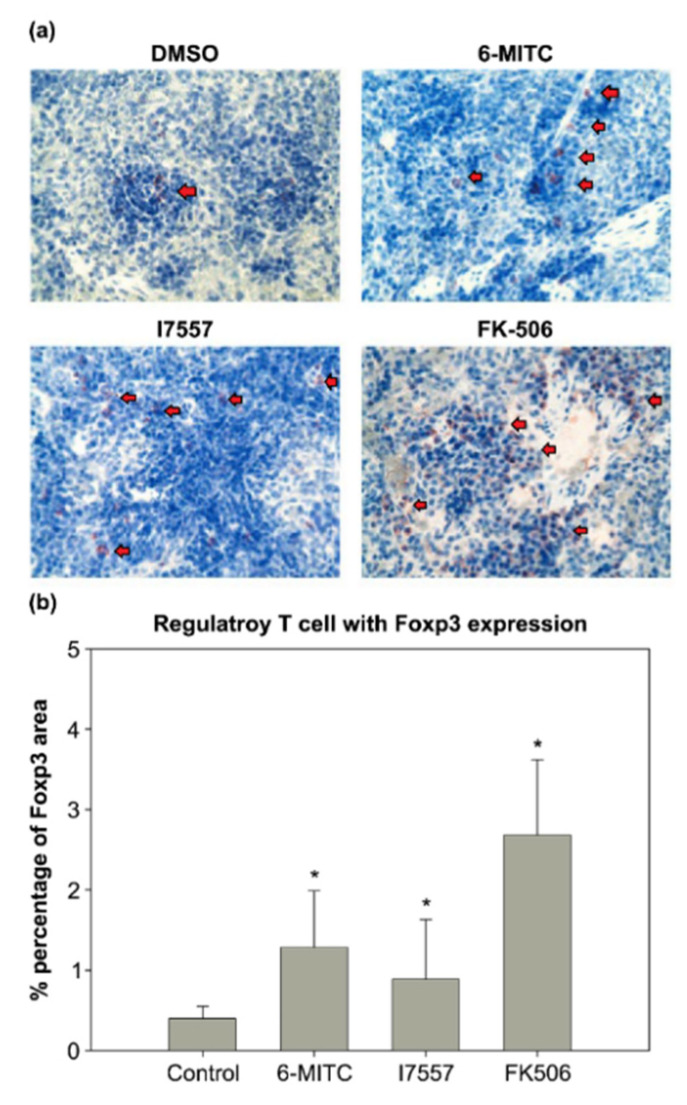
Comparison of spleen tissue histology. The spleen tissue sections were stained for Foxp3 immunohistochemically after treatment with 5 mg/kg 6-MITC, I7557 5, and 3 mg/kg FK-506 compared with the controls. The I7557- and 6-MITC-treated groups exhibited higher regulatory T-cell expression compared with the control group. Arrowheads indicate Foxp3+ cells. (**a**,**b**) In 6-MITC and I7557-treated groups, more Foxp3-expressing Treg cells were accumulated in the spleen * *p* < 0.05 compared with the control group (unpaired Student *t*-test). Magnification 400×.

To examine the effect of 6-MITC and I7557 on tolerogenic APCs, the distribution of Foxp3-expressing T regulatory cells in the recipient spleen was examined. In the control group, the amount of Tregs was decreased and only a few Tregs were found in the spleen (Figure 8). In contrast, in the 6-MITC (5 mg/kg) and 7557-treated (5 mg/kg) groups, more Foxp3-expressing Treg cells were accumulated in the spleen. The percentage of Tregs in the DMSO control, 6-MITC-treated, and I7557-treated groups was 0.40 ± 0.15%, 1.28 ± 0.71, and 0.89 ± 0.74%, respectively.

### 2.9. Evaluation of Hepatotoxicity and Nephrotoxicity

**Figure 9:**
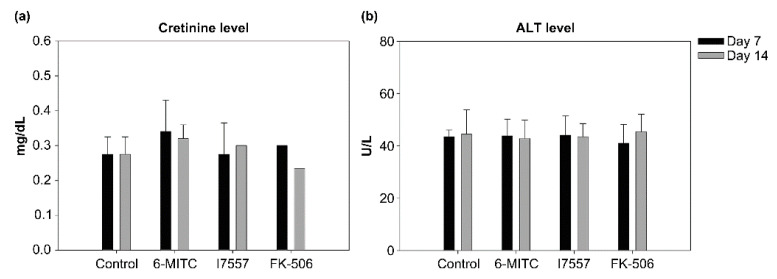
Plasma creatinine (**a**) and alanine aminotransferase (ALT) (**b**) levels of 6-MITC and I7557-treated rats compared with that of the controls following skin transplantation. There were no significant differences in either variable between these groups (*n* = 5 in each group).

After skin transplantation, there were no significant differences between the control, 6-MITC, I7557, and FK506-treated rats in either the ALT or creatinine levels (Figure 9).

## 3. Discussion

The wasabi compound, 6-MITC, and its chemical derivative, I7557, induced human DC differentiation toward a tolerogenic phenotype. These two agents prolonged rat skin allograft survival accompanied by increased Tregs in the spleen, and exhibited no major liver or kidney toxicity.

The transplantation of allogeneic skin grafts can promote a strong immune response that leads to acute rejection [18,19]. This model inevitably results in a destructive and strong immune response triggered immediately after graft transplantation [20]. Several studies indicate that dendritic cells can modulate graft immunotolerance [21,22,23]. Using a skin transplantation model for drug screening may be more predictive compared with other models.

Given that the DCs are the most potent APCs that function in disease, such as autoimmune diseases and transplantation, the wasabi compounds inhibited the maturation of the DCs and induced a tolerogenic phenotype, which may result in their anti-rejection effect. The results of a skin allograft model demonstrated the anti-rejection capacity of wasabi compounds associated with an increase in splenic Tregs. Compared with the positive control, FK-506, the effects on the prolongation of the skin allograft survival and increased splenic Tregs were similar. Whether the mechanism of action of the wasabi compounds resembles that of FK-506 in inhibiting the calcineurin activity remains to be determined.

In our previous studies, we observed the structure activity relationship (SAR) between the wasabi compounds [11,13]. For the treatment of cancer cells, the profile in the potency of wasabi compounds is different from that of the DCs. Against oral cancer SAS cells, the most potent compound was I7557, followed by I7447. In pancreatic cancer cells, the most potent compound was 6-MITC, followed by I7557. In the present study, the most potent compound was I7557, followed by 6-MITC. This indicates that SAR may have differential profiles, depending on the experimental models used.

The dual effect of the wasabi compounds on the allo-stimulatory activity and the cytokine release is intriguing. This type of dose-dependent dual activity is not uncommon in the pharmacologic and biological activities of naturally occurring compounds [24]. For example, arsenic trioxide induced granulocytic differentiation and cell death at lower and higher concentrations, respectively [25]. To our knowledge, this is the first study to demonstrate the dual effects of the wasabi compounds on immune effectors.

Maintenance immunosuppression is best achieved by combination therapy to minimize the side effects of each drug and to maintain immunosuppression by targeting multiple steps of T-cell activation [26]. The CNI dosage should be tailored to prevent nephrotoxicity, infection, and de novo malignancy; thus, 6-MITC and its derivatives may overcome CNI-related side effects and induce tolerogenic dendritic cells for immunotolerance.

One limitation to this study is that no molecular mechanism was addressed. This issue should be addressed and will be assessed in future studies, including transplantation models of solid organs.

## 4. Materials and Methods

### 4.1. Reagents

The compounds 6-MITC, I7447, and I7557 were obtained from LKT Laboratories (St. Paul, MN, USA), dissolved in dimethyl sulfoxide (DMSO) (Merck, Darmstadt, Germany) as a stock solution, and stored at −20 °C until use.

### 4.2. Generation of Dendritic Cells

The human peripheral blood mononuclear cells were obtained from healthy donors, and human dendritic cells were generated as described previously [27]. The CD14^+^ cells were positively selected, using the miniMACS system with anti-CD14 microbeads. The immature DCs were generated from CD14^+^ monocytes by culturing in RPMI 1640 medium supplemented with 10% fetal bovine serum, 5 ng/mL recombinant GM-CSF (R&D Systems, Minneapolis, MN, USA), 5 ng/mL IL-4 (R&D Systems, Minneapolis, MN, USA), every 3 days for 6 days in a humidified 5% CO_2_ incubator. To trigger the maturation of the DCs, immature DCs were incubated with 5 μg/mL lipopolysaccharide (LPS) for an additional 24 h. In these experiments, the 6-MITC, I7457, and I7557 (0, 1, 3.3, and 10 μM) were added at the beginning of the CD14^+^ cell culture, to determine their effects on DC differentiation and maturation.

### 4.3. Morphological Observation

The DCs were centrifuged onto microscope slides using a Cytospin centrifuge (Shandon Inc., Pittsburgh, PA, USA) and then stained with Liu’s A solution for 45 s and Liu’s B solution for 90 s. The DCs were observed under a light microscope (Olympus, Tokyo, Japan) and photographed with a digital camera.

### 4.4. Number of Viable Cells

The DCs were harvested on day 8 and the number of viable cells were counted, using the trypan blue dye exclusion test.

### 4.5. Flow Cytometric Analysis

Dual-color immunolabeling was performed, using fluorescein isothiocyanate (FITC)- and phycoerythrin (PE)-conjugated monoclonal antibodies (mAbs). Mouse anti-human mAbs IgG1: FITC/mouse IgG1:PE along with appropriate isotype controls were purchased from Serotec (Oxford, UK) and used for the DC characterization with anti-CD1a-PE, anti-HLA-DR-PE, anti-CD14-FITC, and anti-CD83-FITC antibodies. After washing twice with PBS, 1 × 10^6^ cells were applied to a FACS-caliber flow cytometer (BD Biosciences, San Jose, CA, USA). The data were collected and analyzed using CellQuest Software (BD Biosciences).

### 4.6. Allogeneic Naïve T-Cell Proliferation and Cytokine Secretion

To purify the CD4^+^CD45RA^+^ T cells, nonadherent cells from the culture of isolated mononuclear cells were used. The naive T cells were enriched using a CD4^+^CD45RA^+^ T-cell isolation kit (Miltenyi Biotec, Bergisch Gladbach, Germany) and a MiniMACS system with magnetic Abs with a negative selection technique. The monocyte-derived 6-MITC- or I7557-treated DCs were harvested and irradiated (30 Gy) with 6 MeV X-ray generated by a linear accelerator (ClinacR 1800, Varian Associates, Inc., Palo Alto, CA, USA), at a dose of 4.0 Gy/min in a single fraction. The irradiated DCs were incubated with 1 × 10^6^ allogeneic naïve T cells at ratios of 1:10 or 1:30 for 5 days, after which 5 μM carboxyfluorescein succinimidyl ester (CFSE) was added to T-cell cultures for 18 h. The cells were then collected, and the incorporated CFSE was detected using flow cytometry.

### 4.7. Detection of Cytokines Produced by DC and Simulated Allogenic Naïve T cells

The levels of IL-10, IL-12, TNF-α, and TGF-β in the DC supernatant, and IFN-γ in the stimulated allogeneic T-cell supernatant were measured using an enzyme-linked immunosorbent assay (ELISA) (R&D Systems), according to the manufacturer’s instructions.

### 4.8. Animals

Male Wistar (donor, 200–250 g) and Sprague Dawley (SD, recipient, 200–250 g) rats were purchased from the LASCO Co. (Taipei, Taiwan). They were housed in a specific pathogen-free environment in compliance with the regulations in the Mackay Memorial Hospital (MMH) Guide for the Care and Use of Laboratory Animals. All of the experiments were approved by the Experimental Animal Committee of MacKay Memorial Hospital, Taipei, Taiwan (MMH-A-S-104-28).

### 4.9. Skin Transplantation Model

A 2 × 2 cm full-thickness skin graft was removed from the flank of a euthanized Wistar donor rat and the underside was gently scraped with a scalpel to remove fat and muscle. The backs of the anesthetized SD recipients were shaved and washed with 70% ethanol. A graft bed was prepared with fine scissors by removing an area of the epidermis and dermis down to the level of the intrinsic muscle. The graft was fixed to the graft bed with eight interrupted sutures of 4-0 monofilament nylon thread (Dermalon; Davis and Geck, St. Louis, MO, USA). No dressings or antibiotics were used. Skin graft survival was evaluated three times a week by visual and tactile examination. The percentage of viable grafts was assessed by calculating the graft viable area.

### 4.10. Drug Preparation and Treatment

The 6-MITC and I7557 were dissolved in dimethyl sulfoxide (DMSO) as a stock solution. Twenty rats were randomly assigned to four groups (five rats each) and treated according to one of the following regimens: (1) DMSO (as a vehicle control); (2) 5.0 mg/kg 6-MITC i.p. per day; (3) 5.0 mg/kg I7557 i.p. per day; (4) 3.0 mg/kg Prograf (FK506) i.p. per day. All of the treatments were started on the day of transplantation and continued until rejection was observed. Post-transplant blood samples were obtained from the tail vein. All of the rats were sacrificed by CO_2_ euthanizing on day 14 after the operation and blood samples were collected by cardiac puncture. Skin grafts and spleens were harvested following sacrifice.

### 4.11. Histological Evaluation

For histologic analysis, the skin grafts were fixed in formalin and embedded in paraffin. The sections (5 μm) were cut, deparaffinized, rehydrated, and stained with hematoxylin and eosin.

### 4.12. Immunohistochemical Analysis

The sections (5 μm) were cut from representative tissue blocks, deparaffinized by rinsing with xylene, and rehydrated in distilled water through a graded alcohol series. For the immunohistochemical analyses, the spleens were fixed in formalin and embedded in paraffin. Based on the histological analysis of skin grafts, the groups treated with 6-MITC 5 mg/kg and I7557 5 mg/kg, FK506 3 mg/kg, and the DMSO groups were evaluated. The antigen retrieval was completed using sodium citrate buffer (pH 6.0). The slides were incubated with anti-FoxP3 primary antibody (Abcam, Cambridge, UK) and detected using the ABC-HRP kit (Vector Laboratories, Newark, CA, USA). For the detection of the forkhead/winged helix transcription factor 3 (Foxp3), a procedure was performed, as described previously.

### 4.13. Evaluation of Leukocyte Count, Hepatic, and Renal Function

The white blood cell counts of the blood samples were analyzed with an automatic Coulter counter (Model Z1, Beckman Coulter Electronics, Fullerton, CA, USA) and the plasma levels of alanine aminotransferase and creatinine were measured by a standard colorimetric method, using manufacturer-supplied reagents.

### 4.14. Statistical Analysis

The results were expressed as the mean ± standard error of the mean (SEM). Comparison of the means was performed by an unpaired Student’s *t*-test, using SPSS software version 12.0 (SPSS, Chicago, IL, USA). A one-way analysis of variance (ANOVA) was used to compare the skin graft survival among groups. The *p* values less than 0.05 were considered statistically significant.

## 5. Conclusions

Overall, 6-MITC and I7557 redirected human DC differentiation and maturation toward a tolerogenic phenotype and promoted regulatory T-cell expansion. The 6-MITC and I7557 effectively prolonged skin allograft survival and have potential as effective immunosuppressants.

## Data Availability

Not applicable.
